# Progress in Clinical Magnetocardiography: The Contactless Breakthrough for Noninvasive Clinical Detection of Cardiac Ischemia Now Needs Worldwide Standardization

**DOI:** 10.3390/s26041369

**Published:** 2026-02-21

**Authors:** Riccardo Fenici, Marco Picerni, Peter Fenici, Donatella Brisinda

**Affiliations:** 1 School of Medicine and Surgery, Catholic University of the Sacred Heart, 00168 Rome, Italy; peter.fenici@unicatt.it; 2 Biomagnetism and Clinical Physiology International Center, 00144 Rome, Italy; mpicerni@sissa.it; 3 International School for Advanced Studies (SISSA), 34136 Trieste, Italy; 4 Fondazione Policlinico Universitario Agostino Gemelli, IRCCS, 00168 Rome, Italy

**Keywords:** magnetocardiography, SQUID, OPM, myocardial ischemia, coronary artery disease, chest pain, ischemic heart disease, noninvasive multimodal imaging of cardiac electrophysiology, magnetoionography, standardization

## Abstract

**Highlights:**

**What are the main findings?**

- A novel technology, optically pumped magnetometers (OPMs) (favoring clinical trials on a large population), has provided substantial evidence supporting the superior performance of rest magnetocardiography (MCG) in the detection or exclusion of ischemic heart disease, compared with rest and even stress ECG.
- A coordinated international and interdisciplinary effort is needed to standardize magnetocardiography for clinical use.

**What are the implications of the main findings?**

- With appropriate standardization, OPM-based MCG could become a quick (and cost-effective) first-level examination approach for the earlier detection of or to rule out signs of myocardial ischemia, especially for patients with chest pain from suspected acute coronary syndrome (ACS) or ischemia with non-obstructive coronary arteries (INOCA), but no diagnostic high-sensitivity troponin or ECG patterns.
- The establishment of an interdisciplinary expert commission is now essential to define consensus-based recommendations for MCG clinical use.

**Abstract:**

Magnetocardiography has received regulatory recognition as a contactless, sensitive aid for physicians to diagnose or exclude myocardial ischemia in chest pain patients, with or without coronary obstruction. Such success, however, might not equate to guideline endorsement or proven clinical effectiveness. Moreover, despite its intrinsic advantages, including unrivalled contactless functional imaging of cardiac electrophysiology and a strong potential for multimodal integration with other imaging methods, its clinical adoption remains limited by the lack of internationally recognized standards and guidelines. This Perspective Review article, highlighting the viewpoints of clinical end users, is a call for urgent action to establish an interdisciplinary expert commission. This is essential for defining consensus-based standards and recommendations for the clinical use of MCG.

## 1. Introduction

Magnetocardiography is the contactless recording of the magnetic field (MF) generated by the electrophysiological activity of the heart, using highly sensitive magnetic sensors placed outside the body. It is a branch of biomagnetism, an interdisciplinary scientific field with theoretical and experimental foundations and development which were discussed and published in the Proceedings of International Workshops, starting with the meeting held in Berlin in 1980 [1], as well as in the NATO Advanced Study Institute [2] and the Biomagnetism Workshop [3], both held in Rome in 1982.

After years of experimental studies in humans using single- or few-channel recording systems, true clinical MCG became feasible with the development of multichannel instrumentation, which enabled real-time, simultaneous multipoint mapping and investigation of cardiac MF dynamics.

For almost forty years, the body of knowledge on clinical MCG applications has been based primarily on studies performed using bulky cryogenic superconducting quantum interference device (SQUID)-based, multichannel MCG mapping systems, mostly operated in magnetically shielded rooms (MSRs) [4] and in unshielded hospital environments [5]. However, despite decades of experimental and clinical research using cryogenic sensor technology [6,7,8,9,10], which demonstrated its potential advantages and diagnostic utility for a wide range of cardiac abnormalities [11,12,13,14,15,16,17,18,19,20,21,22], MCG achieved its first regulatory recognition as a clinical diagnostic tool only after the industrial production of innovative mapping systems based on compact optically pumped magnetometers (OPMs) technology. These systems operate within compact electromagnetic cylindrical shields, into which the patient is slid for the short MCG acquisition time (approximately 90–180 s) [23,24,25]. Such shielding solutions are smaller and less expensive than dedicated MSRs [26]. A similar approach has recently been adopted in China, where a limited number of OPM-based devices for clinical cardiac MF mapping have entered industrial production [16,27,28].

Before the availability of OPM technology, the only officially recognized clinical application of MCG was fetal MCG (FMCG), which is acknowledged as a valuable diagnostic tool for prenatal assessment and monitoring of fetal arrhythmias and the risk of sudden death in pregnancies complicated by high-risk congenital heart disease or channelopathies [29,30,31,32]. FMCG studies were predominantly conducted using SQUID magnetometers in magnetically shielded rooms (MSR [33], but also in unshielded environments [34]. The introduction of OPM-based multichannel systems has significantly simplified clinical implementation. Dedicated instrumentation employing a “person-sized shield” is currently under advanced development [33,35].

A third MCG application of significant diagnostic interest, although not yet officially recognized, is for the three-dimensional (3D) electro-anatomical imaging (EAI) of cardiac electrophysiology, based on inverse solutions of cardiac magnetic fields. This approach has pioneered the concept of pre-interventional noninvasive localization and EAI of arrhythmogenic substrates and is foreseen as enabling advanced noninvasive electrophysiological assessment at a quasi-tissue level [36,37,38].

Notably, recent literature reports have demonstrated a high diagnostic accuracy in OPM-based MCG mapping systems for the diagnosis of ischemic heart disease (IHD). Furthermore, compelling evidence indicates that the diagnostic accuracy of MCG for IHD [39,40,41,42] is not only superior to that of resting ECG, but may approach that of second-level standards of care (SOC) imaging tests, which typically require longer hospitalization and involve risks associated with provocative testing, contrast agents, or ionizing radiation.

However, despite previous attempts [2,3], no universally accepted guidelines or standardized protocols have yet been established for MCG signal acquisition, preprocessing, segmentation, data analysis, nor recommendation for clinical application. Moreover, although basic recommendations for high-resolution MCG recording and post-processing have been discussed since the 1980s [43,44,45,46], clear information regarding MCG signal acquisition parameters (e.g., analogue bandwidth and/or digital sampling frequency) is often omitted or only partially reported in several recent clinical publications [25,28,47]. Furthermore, the reported high-frequency cutoff is commonly limited to 100 Hz, and a wide variety of filtering approaches are employed during post-processing to improve the MCG signal-to-noise ratio (SNR), sometimes reducing the effective analytical bandwidth to as little as 0.1–40 Hz [48].

Although such filtering strategies may produce signals that are more stable and less affected by major artefacts, they may be questionable for diagnostic purposes. By altering both high- and low-frequency components of the original cardiac signal, these approaches can introduce measurement errors in MCG-derived parameters [49] and compromise the unique capability of MCG to provide advanced noninvasive assessment of human cardiac electrophysiology [38].

Moreover, direct comparisons of MCG measurements acquired in the same subjects using multichannel systems based on different sensor technologies and geometric configurations have rarely been performed [50,51], and remain particularly limited for the most recent OPM-based instrumentations [35].

Instead, from the clinical cardiologist’s perspective, MCG must adhere to requirements and recommendations analogous to those established years ago for the digital acquisition and post-processing of electrocardiographic signals [49,52,53,54]. This guarantees immediate readability and full comparability of results, regardless of the equipment used for clinical diagnostic purposes.

This Perspective Review article, drawing on decades of experimental and clinical research using SQUID-based MCG mapping and the reading of the most recent MCG literature, aims to highlight, from the point of view of the clinical end user, the technological requirements for modern, multipurpose MCG systems. Moreover, it seeks to propose recommendations for methodological standardization, consistent with the enhanced diagnostic potential of MCG. In addition, it suggests updating tools for transforming MCG recordings acquired with different sensor technologies into a unified, normalized data format, facilitating immediate data availability and multicenter clinical trials [55,56,57]. Such recommendations may also steer the design of next-generation MCG devices based on emerging technologies, promoting cost-effective scalability, clinical acceptance, and regulatory approval of multimodal, noninvasive functional imaging of cardiac electrophysiology at the patient’s bedside.

## 2. New Technology for Clinical Magnetocardiography

### 2.1. MCG Sensors

Twenty years ago, multichannel SQUID-based systems were the only reliable instrumentation available for mapping cardiac magnetic fields in clinical environments. However, the complexity of system management, high costs, the need for specialized personnel, and the requirement for large and expensive MSRs confined cryogenic SQUID-based MCG to a limited number of highly specialized centers, thereby restricting widespread clinical experience for decades.

The recent commercial availability of highly sensitive and reliable non-cryogenic magnetic sensor technologies represents a major milestone for the large-scale clinical application of MCG.

A detailed discussion of the state of the art in novel magnetic sensor technologies and denoising methods is beyond the scope of this paper. It can be found in several recently published review articles [40,47,58,59,60,61,62]. Therefore, this work focuses solely on selected aspects that, from a clinical perspective, are considered essential for the correct and effective application of MCG in routine clinical practice.

#### 2.1.1. Zero-Field OPM Sensors for MCG Medical Devices

From the perspective of the clinical end user, recently developed MCG mapping systems based on zero-field OPM sensors represent the most reliable alternative to traditional SQUID-based multichannel systems. Although these systems still require the patient to be positioned within a compact cylindrical electromagnetic shield during data acquisition, they avoid the need for the large, dedicated spaces required by cryogenic SQUID-based installations. Operating at room temperature, OPM-based instrumentation does not entail the operational complexity, specialized technical personnel, or high maintenance costs associated with cryogenic MCG systems.

**Figure 1:**
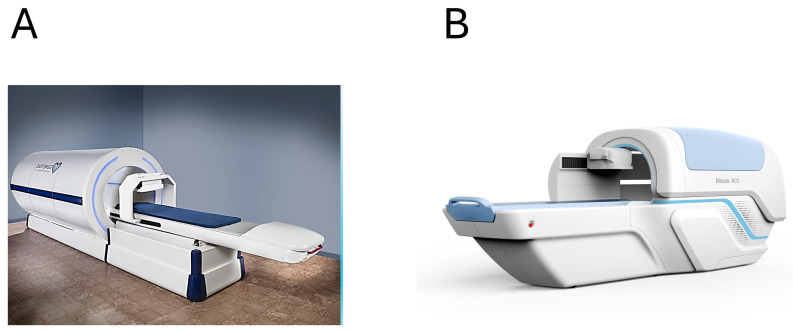
Example of modern OPM-based 36-channel MCG devices: (**A**) The Genetesis CardioFlux^®^. (**B**) The Miracle MCG (X-MAGTECH Technologies Ltd., Beijing, China).

The industrial production of OPM-based devices has markedly simplified their installation and routine use in clinical environments, thereby facilitating clinical trials and the validation processes required for regulatory approval. The OPM-based CardioFlux^®^ system, Genetesis, Mason, OH, USA, (Figure 1A) received its first Breakthrough Device Designation from the U.S. Food and Drug Administration (FDA) in 2020 for the diagnosis of myocardial ischemia and infarction in patients presenting with symptoms suggestive of acute coronary syndrome, followed by a second designation in 2023 for the noninvasive diagnosis of ischemia with non-obstructive coronary arteries (INOCA). More recently, Health Canada approved CardioFlux^®^ for clinical use to aid physicians in the diagnosis of myocardial ischemia [63]. In addition, a similar OPM-based MCG system specifically designed for FMCG is currently under advanced development in the United States [33].

Several OPM-based MCG systems incorporating cylindrical electromagnetic shielding have also been developed in China. Two 36-channel devices, the Miracle MCG (Beijing X-MAGTECH Technologies Ltd., Beijing, China) (Figure 1B) and the LMCG-36A (Hangzhou Lingci Medical Equipment Co., Ltd., Hangzhou, China), have both been approved as medical devices in China [27,28,64]. 

In addition, preliminary studies have been reported using an OPM-based multichannel vector MCG system developed by Q-MAG Technology. Su et al. described promising results obtained with a compact vector MCG device employing high-sensitivity dual-axis spin exchange relaxation-free (SERF) magnetometers arranged in a sequential scanning configuration, with software-based realignment of the three-dimensional components of the cardiac magnetic field within open-ended electromagnetic shielding. The OPM noise floor is around 25 fT/√Hz (the mean noise floor over 60–70 Hz) in the 1–100 Hz bandwidth, with simultaneous gradiometric subtraction of low-frequency noise along the z-direction [65,66].

These findings are of particular interest because they demonstrate the clinical feasibility of vector MCG, which may provide the enhanced spatial sensitivity required for more advanced noninvasive and multimodal assessment of cardiac electrophysiology [38]. Notably, subsequent studies have shown that the dual-axis magnetometer can be upgraded to enable three-axis measurements by exploiting a dual-resonance effect, achieving magnetic field sensitivities of approximately 40 fT/√Hz in the x- and y-axes and about 50 fT/√Hz in the z-axis [67]. When necessary, cross-axis crosstalk can be compensated for using a recently proposed parametric correction method [68].

#### 2.1.2. Scalar OPM Sensors

Since the optimal sensitivity of zero-field OPMs is not yet sufficient for unshielded MCG recordings at the patient’s bedside in noisy hospital wards, alternative approaches have been explored. Although experimental evidence has been reported that SERF-OPM gradiometers could be improved to operate even in unshielded environments [69], efforts have focused on improving the sensitivity achievable with scalar OPMs arranged in gradiometric configurations.

A first generation of Miniature Scalar Atomic Magnetometers (MFAM™, Geometrics Inc., San Jose, CA, USA), originally developed for geophysical applications and operating within the Earth’s magnetic field with a sensitivity better than 2 pT/√Hz up to approximately 400 Hz, was successfully tested in a first-order gradiometric configuration in the unshielded biomagnetic cardiac catheterization laboratory of the Catholic University Hospital in Rome [70]. Fenici et al. reported good agreement between MFAM-based MCG and SQUID-based MCG waveforms sequentially recorded in the same healthy volunteers. MFAM OPMs demonstrated sufficient stability to provide an SNR adequate for unshielded clinical evaluation of ventricular depolarization and repolarization, but not of atrial electrophysiology, indicating the need for further improvements in scalar OPM technology for clinical applications [71]. Similar findings obtained more recently with the same sensors in the United States confirmed this limitation [72].

More recently, novel all-optical intrinsic scalar OPM magnetic gradiometers have been developed, with reported sensitivities on the order of 100 fT/√Hz or better. These performance levels suggest their potential suitability for the development of multichannel MCG and MEG mapping systems capable of operating in unshielded environments [73,74]. These and other innovative OPM sensors have already been successfully applied to unshielded magnetoencephalographic recordings [74,75,76,77,78].

Further advancements achieved in China have enabled unshielded beat-to-beat MCG recordings during daily activities, with sufficient sensitivity to investigate effort-induced changes in heart rate and ventricular depolarization–repolarization dynamics using scalar OPMs [79], thereby expanding the potential diagnostic information obtainable from unshielded MCG. More recent studies have provided additional evidence supporting the feasibility of high-sensitivity, real-time MCG using scalar OPMs in the presence of external magnetic field disturbances [80]. Nevertheless, particular attention must be paid to gradient phase and amplitude errors in atomic magnetic gradiometers when designing biomagnetic imaging systems [81].

#### 2.1.3. Other Magnetic Sensor Technologies

In efforts to develop lower-cost multichannel MCG systems for clinical applications, various alternative sensor technologies have been investigated, with particular emphasis on solutions that are portable, reliable, and capable of operating in unshielded hospital environments [71]. Among these, the VitalScan system (Creavo Medical Technologies, Coventry, UK), a truly portable, unshielded MCG device based on low-cost mini-coil magnetic sensor technology, has been successfully used at the patient’s bedside to detect high-resolution MCG parameters. These include fragmentation scores, late QRS scores, and a novel MCG index quantifying the degree of magnetic field rotation (peak rotation score), which have been proposed as markers of ventricular arrhythmogenic risk [82]. A similar mini-coil magnetic sensor approach has also been employed to develop a wearable MCG device for monitoring experimental cognitive workload [83].

Another promising low-cost approach for unshielded multichannel MCG was the first multichannel system based on microfabricated tunnelling magnetoresistance (TMR) sensor technology combined with digital suppression of environmental and sensor noise. Although its sensitivity was sufficient for evaluating ventricular electrophysiology, it remained inadequate for atrial signal assessment [84]. A recent evolution of this concept is the STORM system, which incorporates an array of 42 novel magnetoresistive sensors (Nivio xMR sensor, TDK Corporation, Tokyo, Japan) featuring improved sensitivity and a wide dynamic range [85]. A multistage post-processing pipeline, including digital bandpass and notch filtering, adaptive noise cancellation, signal averaging, and a novel noise reduction technique called Bayesian signal space projection (SSP) achieved an average improvement in MCG signal-to-noise ratio of approximately 35 dB. As a result, the processed MCG signal quality within the 0.1–40 Hz bandwidth approaches that of a standard ECG and is sufficient to reliably detect even the P wave magnetic field.

Finally, nitrogen-vacancy (NV) diamond magnetometers have demonstrated sensitivities in the range of 12–50 pT/√Hz and have been successfully used for experimental recordings of magnetic fields in proximity to biological tissue, such as isolated nerve or muscle action potential [86,87], for invasive close-proximity high-resolution MCG in living rats [88], and, more recently for non-contact MCG of intact rats [89], and of weak magnetic fields induced by ionic currents in mouse corpus callosum axons [90]. The first human MCG recordings with NV diamond sensors have just been reported. Compared with OPMs, NV magnetometers have a smaller sensing volume (about 0.5 mm^3^), which could simplify the construction of a vector MCG device with a gradiometric configuration. However, the reported sensitivity ranged between 6 and 26 pT/√Hz, when measured in shielded, partially shielded, and unshielded environments, is therefore not yet sufficient for reliable and clinically useful MCG recording [91].

### 2.2. Denoising

A key factor in ensuring the reliability of MCG is the effectiveness of the denoising methods employed, which should ideally suppress noise without altering the frequency components that carry clinically relevant electrophysiological information. A recurring limitation in the surveyed literature is the reliance on ‘black-box’ denoising architectures where efficacy is demonstrated phenomenologically rather than derived structurally. While these methods often achieve high metrics on selected datasets, the lack of theoretical explication regarding the underlying filtering mechanism, specifically its behavior in the frequency domain and phase preservation characteristics, prevents a rigorous assessment of their reliability in clinical settings. As clinicians, we do not consider ourselves adequately qualified to perform a comparative technical evaluation of different denoising methods. Therefore, we intentionally avoided such an analysis and limited ourselves to providing a brief overview of the most recent developments.

As with ECG, early approaches to MCG denoising relied primarily on traditional frequency-domain digital filtering techniques [92,93,94]. Although such methods are still widely used, their effectiveness is limited, and they may compromise electrophysiological information when the signal and noise occupy overlapping frequency bands. To address these limitations, alternative techniques have been introduced, including real-time electronic noise subtraction, adaptive filtering based on separate reference channels for noise characterization and removal, and independent component analysis (ICA) [95,96,97,98,99]. In addition to filtering, signal averaging has traditionally been employed, particularly in high-resolution MCG studies [100,101].

More recently, advanced signal decomposition techniques have been applied to MCG denoising, including wavelet transforms, empirical mode decomposition (EMD), ensemble empirical mode decomposition (EEMD), and variational mode decomposition (VMD) [48,68,79,102,103]. Hybrid approaches combining multiple techniques—such as ICA coupled with EEMD [104], as well as integrated denoising frameworks, including the AOA–VMD–WT method (Arithmetic Optimization Algorithm-based VMD with wavelet threshold denoising), have also been proposed to further enhance noise suppression performance [105]. In parallel, active magnetic compensation control techniques, originally developed to improve the performance of magnetically shielded rooms, have been progressively improved [47].

Finally, machine-learning-based automated approaches have demonstrated effectiveness in improving MCG denoising when combined with ICA [106]. More broadly, machine-learning- and deep-learning-based denoising techniques hold considerable promise for enhancing MCG signal processing by enabling a more efficient separation of physiological signals from noise [107] and real-time noise subtraction during data acquisition.

## 3. MCG Diagnosis of Ischemic Heart Disease

Among the numerous potential clinical applications of magnetocardiography identified since the 1990s [6] and more recently revisited [13,108], the majority of research funding and industrial development efforts have been directed toward the creation of innovative devices for the early diagnosis or exclusion of acute coronary syndrome (ACS) in patients presenting with chest pain in emergency departments (EDs), and, more broadly, for the staging and risk stratification of ischemic heart disease.

This focus is readily understandable, given that chest pain represents the second most common complaint among ED patients, and that a substantial proportion of these individuals are classified as intermediate pretest risk due to the diagnostic uncertainty associated with initial cardiac biomarker testing and ECG-based screening [109]. Furthermore, the need for additional evaluation using provocative testing or noninvasive cardiac imaging (NCI) often results in prolonged ED stays or unnecessary hospital admissions, thereby increasing healthcare utilization and costs related to personnel, medical resources, and pharmacological treatment.

In this context, early evidence demonstrating that MCG, a rapid [25], low-cost, noninvasive, and radiation-free technique, can rule out ACS with a predictive accuracy approaching that of second-level provocative or imaging examinations [41] provided a compelling rationale for investment in this area. Such an approach has the potential to improve patient management by enabling timely treatment or safe discharge, while simultaneously reducing ED length of stay and avoiding unnecessary healthcare expenditures [23,110,111].

### 3.1. New OPM-Based MCG Systems for Diagnostic Application

Following the construction of the OPM-based CardioFlux^®^ prototype, several improved device versions were employed in single-center and multicenter clinical trials, all focused on ischemic heart disease. The results of these studies supported the granting of a first Breakthrough Device Designation by the U.S. Food and Drug Administration (FDA) in 2020 for the diagnosis of myocardial ischemia and infarction in patients presenting with symptoms suggestive of ACS, followed by a second designation in 2023 for the noninvasive diagnosis of ischemia with non-obstructive coronary arteries [23,24,112].

In a multicenter, prospective, observational cohort study, Mace et al. evaluated 390 ED patients with suspected ACS and a HEART score ≥ 3, comparing the diagnostic performance of a 90 s MCG acquisition with standard-of-care (SOC) stress testing for the detection of myocardial ischemia. The mean sensitivity and specificity were 66.7% and 57.1% for MCG, respectively, compared with 67.0% and 89.9% for SOC stress testing. Importantly, the mean time to test completion was significantly shorter for MCG (3.18 ± 1.91 h) than for SOC stress testing (22.71 ± 15.23 h). In addition, two case reports derived from the same multicenter study [111] illustrated the clinical value of MCG in chest pain patients with misleading high-sensitivity troponin (hsTn) results. In one case, repeated positive hsTn measurements led to 23 h of observation, whereas MCG rapidly ruled out ACS. In the other, MCG detected ischemic abnormalities despite normal serial ECGs and hsTn levels; invasive coronary angiography subsequently confirmed critical (99%) stenosis of the left main and left anterior descending arteries, requiring coronary artery bypass grafting. In a subsequent comparative study, the same authors concluded that MCG demonstrated sensitivity and specificity comparable to other SOC noninvasive cardiac tests, with the added advantage of avoiding provocative medications or exercise and thus eliminating the risk of inducing myocardial injury or arrhythmias [41]. Ashokprabhu et al. investigated the diagnostic performance of MCG for detecting coronary microvascular dysfunction (CMD) in patients with angina and non-obstructive coronary artery disease (ANOCA), using coronary flow reserve (CFR), measured invasively or noninvasively, as the reference standard. When invasive CFR was used as the reference, MCG achieved a receiver operating characteristic (ROC) area under the curve (AUC) of 0.66, with a sensitivity of 68% and specificity of 65%. In the subgroup assessed using Doppler-derived CFR, MCG demonstrated improved diagnostic accuracy, with an ROC AUC of 0.76, sensitivity of 75%, and specificity of 77% [24]. The utility of MCG for evaluating obstructive coronary artery disease (CAD) before and after percutaneous coronary intervention (PCI) has also been reported [113].

The diagnostic advantages of OPM-based MCG are further supported by numerous large-scale, multicenter, and interdisciplinary studies conducted in China [28,40,47,114,115,116,117]. For example, Zhang et al. studied 112 patients presenting with chest pain and compared MCG with single-photon emission computed tomography (SPECT). Of 65 automatically derived MCG parameters, 5 were selected and analyzed using three machine learning models to detect impaired myocardial perfusion. Compared with SPECT, all three models demonstrated excellent diagnostic performance, with AUCs of 0.796, 0.780, and 0.804, respectively, characterized by high sensitivity but comparatively low specificity [28]. A well-documented case reported by Ma et al. demonstrated the clinical utility of OPM-based MCG for rapid, reliable, and noninvasive risk stratification in a patient with recurrent angina following PCI. MCG findings indicative of myocardial ischemia normalized after PCI of a subtotal occlusion of the first diagonal branch (D1), but reappeared one month later when angina recurred, despite normal ECG and troponin levels. Repeat coronary angiography confirmed D1 restenosis, which was successfully treated with stent implantation, leading to symptom resolution and normalization of MCG findings during follow-up [118]. Importantly, several OPM-based studies have confirmed the high sensitivity of MCG for detecting or excluding clinically relevant myocardial ischemia when compared with fractional flow reserve (FFR), as originally reported by Park et al. [119]. Yang et al. evaluated 141 adults to develop and validate an OPM-based MCG diagnostic model for detecting myocardial ischemia in patients with borderline coronary lesions before and FFR assessment. The final model, incorporating five MCG parameters, achieved an area under the receiver operating characteristic curve (AUC) of 0.864 (95% CI: 0.803–0.925), with a sensitivity of 79.4%, specificity of 80.8%, a positive predictive value (PPV) of 79.4%, and negative predictive value (NPV) of 80.8% [40], demonstrating the potential utility of OPM-based MCG for functional assessment of coronary stenosis. In a more recent study, Liu et al. compared pre- and post-PCI MCG recordings from 363 patients with non-ST-segment elevation (NSTE) ACS, including 134 patients with post-PCI residual angina, defined as a Seattle Angina Questionnaire—Angina Frequency (SAQ-AF) score < 100 at the 3-month follow-up. Among 65 calculated MCG features, five delta parameters were selected after repeated cross-validation using the least absolute shrinkage and selection operator (LASSO) and were included in the evaluation model, demonstrating strong diagnostic performance, with an average AUC of 0.78 [116]. The authors concluded that “*the developed model, based on dynamic changes in MCG parameters, provides a reliable and clinically meaningful tool for detecting myocardial ischemia and for guiding pharmacological treatment and follow-up strategies in post-PCI patients, thereby improving the management of this high-risk population*”. Wu et al. assessed the accuracy of MCG for detecting coronary artery stenosis in 587 patients by comparison with coronary computed tomographic angiography (CCTA) and coronary artery calcium scoring (CACS). The ROC AUC of MCG for detecting ischemia was 0.80, with an overall sensitivity of 74.6% and specificity of 84.9%. Notably, in patient subgroups with increasing CACS values, the diagnostic specificity of CCTA declined markedly (78.6% vs. 24.1% vs. 17.5%), whereas the specificity of MCG remained relatively stable (92.9% vs. 86.2% vs. 82.5%). When CACS was ≥ 400, the diagnostic accuracy of MCG and of a combined diagnostic model exceeded that of CCTA alone [107]. Furthermore, a recent OPM-based MCG exercise stress test study confirmed the feasibility and clinical value of an approach previously explored using SQUID-based unshielded MCG systems [120,121].

Comparable diagnostic performance was also reported in recent studies employing unshielded SQUID-based MCG mapping systems [122,123,124,125]. He et al. evaluated 291 patients with coronary artery stenosis (30–90%) confirmed by CCTA and CT-derived fractional flow reserve (CT-FFR; cutoff ≤ 0.8). Patients with stable coronary artery disease (SCAD) exhibited significantly higher mean MCG scores than those without SCAD (5.6 ± 2.9 vs. 2.0 ± 1.9; *p* < 0.001), with an ROC AUC of 0.824. The sensitivity, specificity, PPV, NPV, and overall accuracy of the MCG score were 69.6%, 87.9%, 72.7%, 86.2%, and 82.1%, respectively, demonstrating superior specificity and moderate sensitivity compared with CT-FFR [122]. Tolstrup et al. studied 133 patients with acute or chronic chest pain and 63 healthy controls using unshielded SQUID-based MCG. Ischemic chest pain was diagnosed in 41% of patients using SOC noninvasive and invasive testing. An abnormal resting MCG was strongly associated with ischemic chest pain (*p* < 0.0001), yielding sensitivity, specificity, PPV, and NPV values of 86%, 80%, 75%, and 89%, respectively. By comparison, stress SPECT demonstrated sensitivity, specificity, PPV, and NPV of 93%, 72%, 77%, and 91%, respectively [123]. Hunter et al. explored the diagnostic potential of a novel unshielded vector MCG device in three patients with Takotsubo cardiomyopathy, highlighting the potential utility of MCG in stress-induced cardiomyopathies [124]. Finally, a single-center study by Dischl et al., using a 64-channel SQUID-based MCG system, provided preliminary evidence that a novel MCG-derived technique termed magnetoionography (MIG) may improve coronary artery disease detection by characterizing intracellular ion currents during repolarization. Incorporation of MIG-derived parameters into a stepwise linear discriminant analysis increased sensitivity from 90.3% (MCG alone) to 93.5% and specificity from 76.5% to 85.3% [125]. Additional details regarding MIG methodology can be found in [108].

### 3.2. Significant Statistical Heterogeneity Between Studies

As demonstrated by decades of prior research [7,8,42], and confirmed by the results of recent clinical trials, both shielded and unshielded MCG mapping can noninvasively detect electrophysiological abnormalities generated by ischemia of myocardial fibers from the early stages of the “ischemic cascade,” even in the absence of detectable ECG alterations. This capability helps explain the significant diagnostic value of MCG in ischemic heart disease, particularly as an adjunctive tool for the primary screening of patients presenting to the ED with chest pain of unknown or uncertain origin.

However, recent reviews of studies published up to 2023 have highlighted substantial statistical heterogeneity across investigations [27,39]. This variability is likely attributable to the lack of standardization in MCG instrumentation, acquisition protocols, post-processing procedures, patient selection, and diagnostic criteria. Such heterogeneity remains evident even among the most recent studies employing artificial-intelligence-assisted methods for the automated analysis and classification of large MCG datasets [47,117,126], warranting a cautious interpretation of reported results.

**Figure 2:**
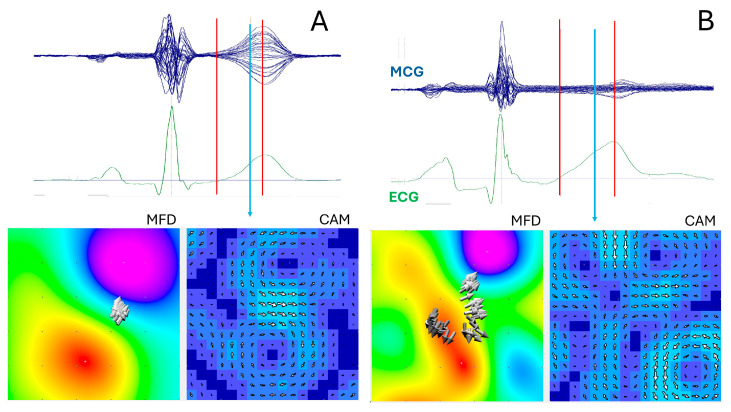
Example of normal ((**A**) healthy subject) and abnormal ((**B**) CAD patient) T-wave magnetic field distribution (MFD) and current arrow map (CAM), computed at the instant marked with the vertical blue arrow. The degree of ventricular repolarization synchronization ((**A**) MFD), and dyssynchrony ((**B**) MFD) is quantified by time-related movement of the equivalent magnetic dipole (solid grey arrows) computed during the T_onset_-T_peak_ interval (marked with red vertical lines).

Most MCG parameters currently used to identify myocardial ischemia are designed to quantify the well-established differences between the stable, dipolar magnetic field distribution characteristic of normal ventricular repolarization (Figure 2A) and the more disorganized or multipolar magnetic field patterns (e.g., abnormal magnetic field and current distribution angles, and repolarization heterogeneity) observed in patients with varying degrees of acute or chronic ischemia (Figure 2B) [127,128,129,130].

However, similar abnormalities in magnetic field distribution have also been reported in a patient with myocarditis and angiographically normal coronary arteries [42]. Consequently, the development of novel MCG classification methods capable of distinguishing IHD from other cardiomyopathies [14,21,131], particularly acute myocarditis [11,12,16,22], is both necessary and deserving of further investigation to overcome this potential limitation. To the best of our knowledge, however, studies assessing the clinical impact of misclassification in emergency department triage scenarios have not yet been reported.

Indeed, despite cumulative reports of diagnostic accuracy for IHD in the range of approximately 84–85%, with positive and negative predictive values exceeding 90%, even a residual uncertainty of around 10% may raise ethical and medico-legal concerns in real-world clinical practice. This is particularly relevant in the absence of official guidelines from scientific societies and formal regulatory approval for the diagnostic use of MCG, and especially when considering ED discharge decisions for patients with chest pain and an intermediate pre-test probability of IHD.

At present, efforts should be directed toward establishing standardized acquisition and analysis protocols and toward pooling sufficiently large, well-curated, comparable datasets to enable robust statistical evaluation of the most clinically relevant MCG parameters.

Furthermore, analytical strategies should be expanded beyond current approaches to fully exploit the unique capability of MCG to provide multimodal electroanatomical imaging of cardiac current density dynamics.

### 3.3. Multimodal Electroanatomical Imaging of Equivalent Cardiac Current Density in Patients with IHD

Using MCG data, current density imaging (CDI) can be performed by reconstructing the spatial distribution of electrical currents within the heart from measured magnetic fields. This reconstruction typically relies on the resolution of the inverse problem to estimate the equivalent current density based on distributed source models. These models were first formulated by Hämäläinen and Ilmoniemi in 1984 to estimate the brain’s primary current distribution from measured neuromagnetic fields [132].

Distributed source models offer a more physiologically accurate representation of cardiac current sources, particularly when ventricular electrical activation propagates through the myocardium as a wavefront, a condition for which point-like source models are inadequate. The solution of the cardiomagnetic inverse problem using distributed dipolar elements is commonly referred to as the minimum norm estimate (MNE) or current density estimate (CDE).

CDE methods were originally developed and validated to improve the accuracy of three-dimensional localization of focal cardiac sources [133,134,135] and, more broadly, of arrhythmogenic substrates [136]. Since the 1990s, CDE has also been explored for the detection and localization of myocardial ischemia and for the assessment of myocardial viability [137]. In subsequent studies, CDE was computed on the epicardial surface of the left ventricle (LV) from MCG recordings in 13 patients with IHD, acquired before and after exercise-induced acute ischemia [138,139]. Individual torso and LV geometries were reconstructed from magnetic resonance (MR) images. CDE was calculated using second-order Tikhonov regularization applied to ST-segment MCG data, obtained as the difference between resting and post-exercise measurements. In patients with single-vessel coronary artery disease, increased CDE amplitude correlates with the expected ischemic myocardial regions supplied by the stenotic coronary artery. In patients with three-vessel CAD and regional or global LV dysfunction caused by severe chronic ischemia, prior myocardial infarction, or both, the same CDE approach was applied and compared with positron emission tomography (PET) imaging. PET was used to differentiate viable myocardial tissue from scar regions, displayed on the same eight-sector LV polar map subdivided into three short-axis planes (apical, mid-ventricular, and basal). Diagnostic agreement was defined by the presence of low CDE amplitude in PET-defined scar segments or high CDE amplitude in viable segments. An average correspondence between CDE-derived regions with high and low CD amplitude and the PET-defined viable and scar areas, respectively, was observed in 77% of patients.

In a study by Nakai et al. [140], three-dimensional current density was reconstructed from 64-channel MCG data, using the magnetic field component orthogonal to the anterior chest wall (Bz), employing a spatial filter and Tikhonov regularization. Both atrial and ventricular outlines were derived from the integrated current density images. Compared with healthy controls, patients with prior myocardial infarction (MI) exhibited longer dispersion maps of corrected ventricular recovery time and prolonged negative dT/dt at the T-wave peak–end interval. When superimposed on the cardiac outlines generated by MCG, these abnormalities corresponded spatially with MI locations identified by Tc-99m tetrofosmin myocardial perfusion imaging. The diagnostic value of three-dimensional current density dispersion alternans assessed by MCG mapping in patients with IHD and ventricular arrhythmias was further confirmed in a more recent study [141].

Conversely, a study evaluating the clinical utility of various MCG-based current density reconstruction methods for myocardial viability assessment, using PET as the reference standard, reported insufficient diagnostic performance when CDE was computed from resting MCG recordings alone. The authors concluded that vector MCG measurements, *the inclusion of stress testing, and further advancements in mathematical modelling could substantially improve CDI performance* [142].

To minimize errors in three-dimensional localization and imaging of cardiac sources, an end-diastolic torso–heart model should be employed for source reconstruction during ventricular depolarization. In contrast, source reconstruction during the T wave is susceptible to substantial inaccuracies unless a systolic torso–heart model is used [143]. Ultimately, optimal electrophysiological imaging may be achieved by synchronizing and integrating four-dimensional MRI-based anatomical imaging of the individual patient’s heart with real-time, functional MCG data [139].

## 4. Recommendations for MCG Standardization

### 4.1. Digital Recording and Postprocessing of the MCG Signal

Since the early 1980s, MCG signal acquisition, post-processing, and waveform analyses have been performed digitally to fully exploit the potential of contactless magnetocardiography, enabling high-resolution waveform analysis and body-surface mapping of electrophysiological events beyond what is achievable with conventional ECG recordings [45,144,145]. Digital processing of MCG signals begins with the sampling of an extremely weak analog signal recorded by magnetic sensors, followed by digital denoising procedures aimed at improving the SNR. These procedures suppress patient-related low-frequency noise, primarily due to respiration and body movements, high-frequency noise associated with muscle activity, power line interference, and other sources of environmental electromagnetic noise.

Despite these long-standing advances in digital MCG processing, data acquisition protocols, including hardware configurations, recording bandwidths, and sampling frequencies, remain insufficiently standardized.

#### 4.1.1. MCG Recording Bandwidth and Sampling Frequency

Regardless of the ECG lead configuration or the MCG sensor position, both ECG and MCG waveforms exhibit the same characteristic P–QRS–T pattern, corresponding to atrial depolarization (P wave), ventricular depolarization (QRS complex), and ventricular repolarization (T wave). Consequently, established ECG recommendations should also be applied to the digital acquisition and post-processing of MCG signals [53,54,146].

Early spectral analyses of adult ECG signals suggested that most diagnostic information was contained below 100 Hz. However, subsequent studies demonstrated that an upper bandwidth cutoff of at least 150 Hz is required to limit waveform amplitude estimation errors to less than 25 µV in 95% of adult and adolescent subjects, while an upper cutoff of 250 Hz is recommended for pediatric ECG recordings [49]. In addition, high-resolution analyses of the QRS complex have revealed diagnostically relevant high-frequency components, particularly in pathological conditions [147,148,149,150].

An upper frequency cutoff below 50 Hz, often used in recent studies, might result in excessive smoothing and inaccurate quantitative assessment of MCG amplitude parameters, thereby compromising diagnostic performance, especially in younger patients. Because high-frequency components characterize the most rapidly changing portions of the signal, inadequate high-frequency bandwidth may systematically smooth notched features within the QRS complex that are clinically relevant, for example, in the high-resolution detection of abnormal fragmentation associated with arrhythmogenic cardiomyopathy [147,151,152,153,154]. Accordingly, as early as the 1980s, a 250 Hz upper-frequency cutoff for the analogue input signal was recommended as a standard requirement for both surface ECG and MCG recordings of cardiac electrophysiological signals [45].

Similarly, the low-frequency cutoff of the analog ECG signal, initially set at 0.5 Hz to compensate for baseline drift caused by respiration or movement, was subsequently reduced to 0.05 Hz to minimize measurement errors related to excessive low-frequency attenuation, particularly for ST-segment changes induced by exercise or pharmacological stress testing [49].

Moreover, considering the unique capability of MCG to detect potentially arrhythmogenic DC currents, such as those induced by localized myocardial ischemia [9], the recommended bandwidth for MCG signals before digital recording should ideally extend from DC (0 Hz) to 250 Hz, whenever compatible with the characteristics of the magnetic sensors used [6]. For studies involving small experimental animal models, a wider bandwidth (DC–500 Hz, or higher) is preferable [155].

According to the Nyquist theorem [156], the recommended input signal bandwidth of 0.05–250 Hz implies a minimum sampling frequency of 500 Hz. However, since the Nyquist theorem only holds for samplings performed over an infinite time horizon, a higher sampling rate, typically at least 1 kHz, is preferable in practice.

Furthermore, oversampling, defined as sampling at a frequency substantially higher than that required for standard ECG or MCG post-processing, can improve signal fidelity near the high-frequency cutoff and is essential for accurate digital recording of narrow pacemaker pulses, which often have durations shorter than 0.5 ms. Higher oversampling rates (e.g., 5 kHz) also allow more precise capture of fine waveform details, including rapid signal upstrokes and downstrokes [85]. Consistent with this concept, a recent ECG work combining conventional negative-derivative ECG (ND-ECG), which primarily reflects epicardial activation, with ultra-high-frequency ECG (UHF-ECG) has demonstrated the ability to assess activation within a larger volume of the ventricular wall [148]. These findings highlight the diagnostic potential of high-frequency signal components for assessing transmural ventricular activation and support routine oversampling of MCG signals, not only to enhance adaptive digital filtering for noise reduction, but also to expand its diagnostic capabilities, for example, by enabling more detailed analyses of ventricular electrical activation or the assessment of pacing-induced interventricular dyssynchrony in patients with MRI-compatible implanted devices [148].

#### 4.1.2. The MCG Coordinate System and the Magnetic Field Color Coding

The Frank Lead System and the MCG Coordinates

The standardized ECG lead coordinate system was originally based on Einthoven’s bipolar limb leads and was subsequently extended to include unipolar limb leads and precordial leads. In 1956, Ernest Frank developed an improved lead system for clinical spatial vectorcardiography (VCG) [157], the polarity of which is consistent with that of conventional ECG leads and body surface potential mapping (BSPM) [158]. The integration of Frank’s X–Y–Z coordinates defines the patient’s frontal (X–Y), axial (X–Z), and sagittal (Y–Z) planes, which are conventionally used in three-dimensional anatomical imaging [159]. For decades, diagnostic interpretation of ECG, VCG, and BSPM has relied on this standardized coordinate system. To preserve this standardization in magnetocardiography and to avoid potential confusion arising from the use of “physicist” XYZ coordinates, which differ in axis orientation and polarity, a joint commission of physicists and cardiologists convened during the NATO Conference on Biomagnetism [2] and the Fourth International Conference on Biomagnetism [3], both held in Rome in 1982, recommended adopting the Frank lead coordinate system for MCG.

Accordingly, for a dipolar cardiac magnetic field distribution generated by an equivalent cardiac current dipole, the Z component of the magnetic field directed outward from the chest produces a negative (downward) deflection in the MCG signal, whereas a magnetic field directed inward toward the chest produces a positive (upward) deflection [160].

The Magnetic Field Color Coding

**Figure 3:**
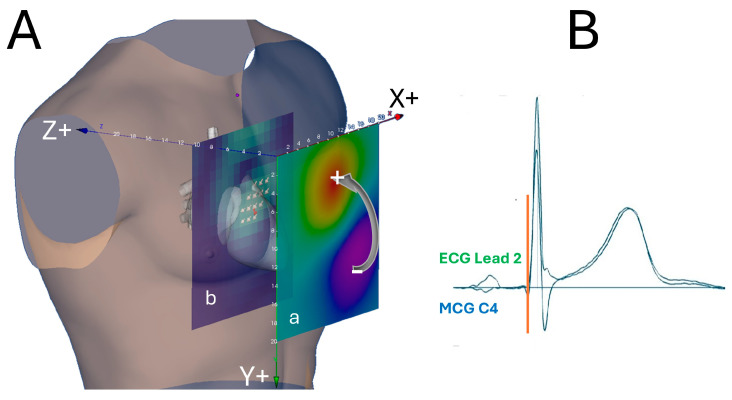
(**A**) Standardized MCG three-dimensional coordinates, defined in accordance with the polarity of the XYZ vectorcardiography Frank lead system. (**a**) Color-coded magnetic field distribution computed at the onset of ventricular depolarization (indicated by the red vertical bar in (**B**)), corresponding to the initial depolarization of the interventricular septum; (**b**) Pseudo–current reconstruction plane intersecting the heart model at the depth corresponding to the localization of the equivalent magnetic dipole source. (**B**) Superposition of ECG lead II (green trace) and MCG recording at position C4 (blue trace).

In accordance with the polarity color conventions used in BSPM, regions of negative magnetic field are coded in blue, while regions of positive magnetic field are coded in red (Figure 3) [6,47].

#### 4.1.3. Methods for the Transformation of Multichannel Magnetocardiographic Signals to Standard Format

A method for transforming multichannel biomagnetic recordings into a standardized spatial grid format, suitable for data pooling and cross-platform comparison, was originally introduced by Numminen et al. [55]. In this approach, the MNE of the distributed cardiac source current density was first computed, and the magnetic field components at predefined standard grid locations were subsequently reconstructed from the MNE using recordings obtained from three subjects with both a 24-channel multichannel system and a single-channel reference instrument. The magnetic signals extrapolated from the multichannel recordings showed good agreement with the single-channel measurements acquired at the standard Finnish grid locations, particularly at positions directly covered by the multichannel sensor array. Subsequently, Burghoff et al. [56] employed a multipole expansion combined with MNE, computed at 1 ms intervals over the cardiac cycle, to compare MCG recordings acquired with two different multi-sensor systems. This method yielded an average correlation of 93% between reconstructed and measured signals, demonstrating the feasibility of sensor-independent normalization of MCG data. More recently, Marhl et al. successfully applied MNE-based transformation and normalization procedures to enable direct comparison of biomagnetic data recorded with conventional SQUID-based and OPM-based MEG systems [57], further supporting the applicability of this approach for standardized data representation and interoperability across sensor technologies.

## 5. Discussion

After decades of research that was largely overlooked by the medical community, magnetocardiography is now experiencing a strong impetus toward clinical recognition. This progress was also facilitated by the recent availability of the OPM-based multichannel MCG devices, developed with the explicit goal of providing a user-friendly, rapidly deployable point-of-care system for intensive use in emergency departments, as well as in peripheral outpatient settings through network connectivity and automated, AI-assisted diagnostic feedback.

**Figure 4:**
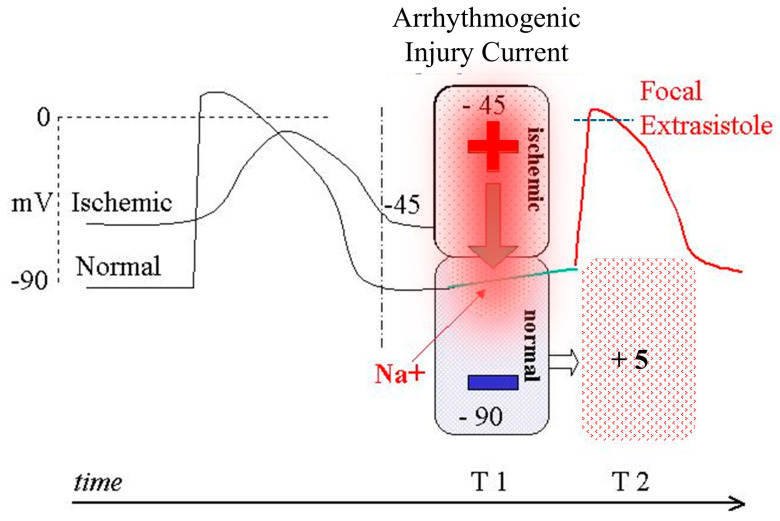
Schematic hypothesis of the potential arrhythmogenic mechanism of an ischemic injury current. During electric diastole (T1), a potential difference exists at the border zone between the partially depolarized ischemic myocardium (−45 mV) and the surrounding healthy myocardium (−90 mV). If the resulting electrotonic current, due to a transitory critical factor (e.g., an autonomic modulation imbalance [167]), reaches sufficient intensity to depolarize the healthy tissue (intracellular red-shaded area and downward pointing thick arrow), it induces the onset of a focal extrasystole (T2), which might initiate a sustained reentry arrhythmia (Modified from [136]).

Efforts aimed at validating this approach through multiple clinical trials have yielded results sufficient to obtain two U.S. FDA Breakthrough Device Designations for the diagnosis of myocardial ischemia in patients with CAD or with INOCA, as well as regulatory approval by Health Canada. Parallel industrial development in China, together with the results of numerous clinical trials, has further strengthened the evidence supporting the diagnostic accuracy of MCG for IHD. However, present evidence that resting MCG outperforms resting ECG, and even stress-ECG, for the early detection of myocardial ischemia, whether related to obstructive CAD or not, might not equate immediate clinical applicability in the absence of guideline endorsement, which requires the definition of consensus-based recommendations and level of evidence for clinical use. Moreover, a fully satisfactory mechanistic explanation for MCG’s higher diagnostic sensitivity is still lacking, although likely arising from its unique direct relationship of cardiac magnetic fields with the primary electrophysiological sources: the intracellular primary currents flowing during cellular depolarization and repolarization [161]; the minimal distortion of the magnetic field induced by inhomogeneities in tissue conductivity and interposed body fluids between the myocardium and the sensors. Another advantage of MCG is the detection of electrically silent magnetic fields, such as potentially arrhythmogenic-ischemia-induced diastolic injury currents [9,162,163], or fields generated by vortex currents, which can be directly measured and quantified using appropriately recorded MCG [164,165,166]. A schematic hypothesis of the potential arrhythmogenic mechanism of the ischemia-related diastolic injury current is proposed in Figure 4. MCG identification and quantitative assessment of such currents before they reach the critical threshold could contribute to preventing life-threatening arrhythmias in ACS patients.

Further insights are provided by recent mathematical modelling studies demonstrating a strong relationship between action potential features and the corresponding temporal and spatial behavior of the cardiac magnetic field. Notably, one such study provided previously unreported evidence that MCG detection of ventricular repolarization alternans is more efficient and “could substitute for, or even outperform,” detection based on conventional electrical parameters [168]. A subsequent study by the same group, investigating how spatially discordant alternan (SDA) of repolarization modulates the cardiac magnetic field, demonstrated that the integrative relationship between the magnetic field and the action potential can amplify subtle alterations of the upstroke (Phase 0), which may not be readily detectable with standard electrophysiological measurements [169]. These findings warrant increased attention to ischemia-related, potentially arrhythmogenic alterations of ventricular depolarization [154,170,171,172].

Additional supporting evidence comes from the clinical study by Dischl et al., which demonstrated that MCG-derived magnetoionography indices, designed to characterize intracellular ion currents during ventricular repolarization, significantly improve MCG diagnostic accuracy for CAD [125]. Although clinical experience with magnetoionography remains limited, preliminary observations suggest that further methodological development could substantially enhance the ability of MCG to noninvasively assess cardiac electrophysiology at a quasi-cellular level, enabling improved diagnostic differentiation between ischemic and non-ischemic cardiomyopathies [108]. Continued advances in MCG signal processing that exploit its unique sensitivity to intracellular electrophysiological phenomena are therefore likely to provide a more complete mechanistic understanding of the superior performance of MCG in the clinical diagnosis of myocardial ischemia.

Nevertheless, despite evidence that wider research work is still needed for a more exhaustive understanding of the mechanisms underlying the MCG’s multipurpose diagnostic potential, recent industrial efforts have been mostly addressed to the construction of MCG devices optimized primarily for a single clinical application, namely the diagnosis of IHD, rather than toward the development of multipurpose MCG platforms suitable for broader diagnostic use. From our perspective, this focus represents a significant limitation to the efficient and cost-effective advancement of MCG instrumentation for clinical practice. This limitation likely reflects insufficient interdisciplinary interaction between device technologists and end users, particularly cardiologists and electrophysiologists with expertise in biomagnetism, who could otherwise provide informed guidance on the technical requirements of a truly multipurpose MCG system.

A key challenge in the clinical translation of magnetocardiography is the absence of internationally shared standards. As previously achieved for electrocardiography, this gap could be addressed through the establishment of an interdisciplinary expert commission charged with evaluating all methodological aspects of MCG and issuing consensus-based recommendations for its clinical use. Given the intrinsic properties of MCG, particularly its ability to facilitate noninvasive inverse estimation of cardiac currents from magnetic field distributions [137,139] and its strong potential for three- and four-dimensional multimodal integration, such standardization is both timely and necessary.

In Section 4, we briefly summarized a limited set of fundamental recommendations, including appropriate recording bandwidth and sampling frequency for MCG signals, adherence to the Frank lead coordinate system, and the use of magnetic-field color coding consistent with BSPM. These recommendations were originally defined by an interdisciplinary expert group in the 1980s to facilitate data pooling, enable meaningful comparisons among research centers, and promote correct interpretation of the relationships between standardized ECG-based techniques and emerging biomagnetic measurements. Despite their continued, and arguably increased, relevance, these principles appear to be often disregarded in current practice [116], and there are no clear guidelines for the clinical operation of OPM-MCG [117].

Several additional issues require targeted investigation before comprehensive standardization can be achieved. For example, based on the currently available literature, the following remains insufficiently defined (beyond theoretical considerations [116]): the extent to which OPM sensor density (i.e., the number of recording channels) and the simultaneous measurement of all three magnetic-field vector components influence the diagnostic performance of OPM-based MCG systems (as would reasonably be expected from prior experience with SQUID-based MCG). Notably, most reported clinical trials for IHD detection rely on recordings acquired with 36-channel OPM systems. Although at least one manufacturer has introduced a 64-channel version of its OPM-based MCG device, comparative performance evaluations have not yet been reported.

These critical aspects should be addressed by an international expert commission to guide focused research where evidence is lacking and to issue recommendations essential for standardizing hardware configurations, post-processing pipelines, and the selection of the most effective diagnostic parameters and algorithms before their use in training AI-assisted automated systems. To ensure comparability among recordings acquired with different sensor technologies, transformation to a common standardized data format should be performed before post-processing and analysis [55,56,57]. Such guidance is mandatory to ensure controlled, transparent, and reliable clinical application.

By contrast, the recent literature on the diagnostic accuracy of OPM-based MCG for IHD reveals a proliferation of parameters with disparate and sometimes “exotic” terminology, even when describing comparable magnetic-field features, which can generate confusion, particularly among clinicians unfamiliar with MCG, despite recent efforts to clarify correlations among different reports [116]. At the same time, diagnostic performance is increasingly delivered by AI-assisted “black-box” systems and is often expected to be accepted without insight into the underlying physiological mechanisms, thereby limiting clinicians’ ability to assess plausibility and correctness independently.

Our perspective is grounded in decades of direct experience confronting the need to manually correct erroneous automated ECG diagnoses and in translating that experience into the development of a user-friendly and reliable MCG system intended for multipurpose diagnostic applications. Importantly, the number of interdisciplinary experts in MCG has now grown substantially, making it both feasible and timely to establish a dedicated international working group to define appropriate, widely accepted standards and recommendations and provide the evidence needed to overcome skepticism [173].

## 6. Conclusions

Notably, this paper is neither a systematic review nor a meta-analysis. Therefore, although we have carefully examined the most recent literature, some relevant contributions, particularly those published in non-medical journals, may have been inadvertently overlooked. Nevertheless, despite the statistical heterogeneity previously reported across studies [27,39], substantial evidence supports significant complementary clinical utility of MCG for earlier detection or exclusion of ischemic heart disease, especially in patients presenting to the emergency department with chest pain of unknown origin and still non-diagnostic hs-Tn levels and ECG patterns. This diagnostic advantage is foreseen to be useful in reducing patients’ ED stay for chest pain management and the need for unnecessary higher-level examinations, undue invasive procedures, and related costs [110,174], while accelerating interventional treatments when indicated [111,113]. However, although MCG is approaching clinical maturity, its broader adoption remains limited by the absence of internationally shared standards and guidelines recommendations. Therefore, as was successfully achieved for ECG, the establishment of an interdisciplinary expert commission is now essential to define consensus-based recommendations for MCG clinical use.

It is particularly urgent to address key unresolved issues that require coordinated investigation, such as defining the impact of sensor density and vector component acquisition on diagnostic performance, and identifying, in greater detail, the fundamental requirements for the technological development of next-generation MCG systems. Such systems should be designed in accordance with the principles of advanced noninvasive functional electrophysiological imaging and optimized multimodal integration of MCG with other noninvasive imaging techniques [175,176,177,178,179,180].

## 7. Future Directions

The time is ripe for a coordinated international effort to standardize magnetocardiography for clinical use. Building on the successful precedent established for electrocardiography, scientific societies, regulatory bodies, clinicians, and physicists should collaborate to form an interdisciplinary commission dedicated to defining consensus-based technical and clinical guidelines for MCG. Such an initiative, certainly useful for the companies engaged in MCG devices development and for the clinical end users, should address hardware configurations, acquisition protocols, post-processing pipelines, parameter selection, validation strategies, and the definition of consensus-based diagnostic protocols, particularly to standardize the mandatory features of emerging OPM-based systems and AI-assisted diagnostic tools.

Standardization is essential to ensure transparency, physiological interpretability, and reproducibility, thereby safeguarding clinical trust and facilitating regulatory approval. Establishing these shared standards will be a decisive step toward unlocking the full potential of MCG as a reliable, noninvasive, and multipurpose modality for cardiac electrophysiological imaging.

## Data Availability

Being a review of the literature (including personal publication of the authors), all data supporting the paper can be found in the 181 cited references.

## Abbreviations

The following abbreviations are used in this manuscript:

MCG	magnetocardiography
SQUID	superconducting quantum interference device
OPM	optical pumped magnetometer
MFD	magnetic field distribution
ACS	acute coronary syndrome
CAM	current arrow map
INOCA	ischemia with non-obstructive coronary arteries
ANOCA	angina and non-obstructive coronary artery disease
SOC	standards of care
SPECT	single-photon emission-computed tomography
CAD	coronary artery disease
CCTA	computed tomographic angiography
IHD	ischemic heart disease
CACS	coronary artery calcium scoring
BSPM	body surface potential mapping
VCG	vectorcardiography
VMD	variational mode decomposition
EEMD	ensemble empirical mode decomposition
EMD	empirical mode decomposition
ICA	independent component analysis
hsTn	high-sensitivity troponin
ED	emergency department
ROC	receiver operating characteristic
AUC	area under the curve
FDA	U.S. Food and Drug Administration
ICA	invasive coronary angiography
CFR	coronary flow reserve
FFR	fractional flow reserve
PPV	positive predictive value
NPV	negative predictive value
PCI	percutaneous coronary intervention
SCAD	stable coronary artery disease
MIG	magnetoionography
CDI	current density imaging
CDE	current density estimate
MNE	minimum norm estimate
PET	positron emission tomography
SNR	signal-to-noise ratio
UHF	ultra-high-frequency
DSE	dobutamine stress echocardiography
LASSO	least absolute shrinkage and selection operator
MNE	minimum-norm estimate
MEG	magnetoencephalography
SDA	spatially discordant alternans
SSP	signal space projection
